# Health-related quality of life of patients with brain metastases selected for stereotactic radiosurgery

**DOI:** 10.1007/s11060-019-03186-z

**Published:** 2019-05-09

**Authors:** Eline Verhaak, Karin Gehring, Patrick E. J. Hanssens, Margriet M. Sitskoorn

**Affiliations:** 1 Gamma Knife Center, Elisabeth-TweeSteden Hospital, Tilburg, The Netherlands; 2 Department of Neurosurgery, Elisabeth-TweeSteden Hospital, Tilburg, The Netherlands; 3 Department of Cognitive Neuropsychology, Tilburg University, Tilburg, The Netherlands

**Keywords:** Brain metastases, Cancer, Gamma knife radiosurgery, Health-related quality of life, Patient-reported outcome measures, Radiosurgery

## Abstract

**Purpose:**

Information on health-related quality of life (HRQoL) of patients with brain metastases (BM) before stereotactic radiosurgery (SRS) is very relevant to improve communication between patients and clinicians and to be able to interpret changes in HRQoL after SRS. The aim of this study was to evaluate the prevalence and severity of complaints on different aspects of pre-SRS HRQoL among patients with BM and to identify predictors thereof.

**Methods:**

Patients with 1–10 newly diagnosed BM, expected survival > 3 months, Karnofsky Performance Status ≥ 70, and scheduled to undergo SRS were included. HRQoL was measured with the Functional Assessment of Cancer Therapy-Brain (FACT-Br) questionnaire. One-sample z-tests were conducted to analyze differences between patients with BM and published normative data of a general adult sample and of an adult cancer sample. Multiple regression analyses were run to identify predictors of pre-SRS HRQoL.

**Results:**

On the individual level, most patients with BM (57.6% of 92 included patients) reported complaints regarding emotional well-being. As a group, patients with BM reported significantly lower emotional well-being compared to both control groups and significantly higher social well-being compared to the general population. Worse psychological factors, e.g. physical fatigue, depression, mental fatigue and anxiety, predicted aspects of pre-SRS HRQoL.

**Conclusions:**

An increased understanding of pre-SRS HRQoL and predictors hereof, provides us with more insight into the well-being of our patients with BM and is necessary for the interpretation of (changes in) HRQoL after SRS.

**Electronic supplementary material:**

The online version of this article (10.1007/s11060-019-03186-z) contains supplementary material, which is available to authorized users.

## Introduction

Often, patients with brain metastases (BM) experience several symptoms prior to treatment of the BM, such as headaches, seizures, focal neurological deficits and cognitive deficits [1–4], which can negatively influence patients’ health-related quality of life (HRQoL). Patients with BM rated HRQoL as the most important factor to be considered in choosing among available treatment options [5]. Since stereotactic radiosurgery (SRS) is increasingly used to treat patients with single or multiple BM [6, 7], evaluating HRQoL of patients selected for SRS has become more important.

Habets et al. [8] were the first to compare HRQoL of patients with BM selected for SRS to the general population. The pre-SRS HRQoL subscale scores of the 97 patients were significantly worse compared to the general population [8]. In a subsequent study, evaluating HRQoL in 55 patients with BM pre-SRS, 89% of the patients had a significantly worse score on at least 1 of the 6 HRQoL scales compared to the general population, with physical functioning (57%) most often affected [9].

Only a few studies investigated predictors of HRQoL in patients with BM. Higher KPS pre-SRS has been associated with better pre-SRS HRQoL [8] and post-SRS HRQoL [10, 11]. In addition, pre-SRS asymptomatic BM, low recursive partitioning analysis (RPA) class, no seizures and no self-reported cognitive impairment were associated with higher HRQoL following SRS [11].

Information on pre-SRS HRQoL is crucial to the interpretation of (changes in) HRQoL after treatment, both for research and clinical practice. The aim of the current study is to evaluate the prevalence of patients with BM scoring clinically meaningful lower on different aspects of HRQoL pre-SRS as compared to the general adult population. In addition, the severity of HRQoL complaints were evaluated by comparing HRQoL scores of patients with BM to normative data of a general adult sample and of an adult cancer sample. The adult cancer sample was used to evaluate the additional effect of being diagnosed with BM. A second aim was to identify potential pre-SRS sociodemographic, clinical, psychological and cognitive predictors of pre-SRS HRQoL.

## Methods

This study evaluates the pre-SRS data from a prospective single-arm study to evaluate cognitive functioning up to 21 months after SRS with Gamma Knife in patients with BM (CAR-Study A; ClinicalTrials.gov Identifier: NCT02953756). Secondary endpoints included patient reported outcomes. The study was approved by the medical ethics committee Brabant (File NL53472.028.15).

### Patients

Patients with BM, scheduled for SRS, were screened in the Elisabeth-TweeSteden Hospital in Tilburg, The Netherlands. Eligibility criteria for SRS were: clinical presentation consistent with BM, contrast enhanced volumetric MRI-scan showing 1–10 newly diagnosed BM, histologically proven malignant primary tumor, lesion ≥ 3 mm from optic apparatus, Karnofsky Performance Status (KPS) ≥ 70, and anticipated survival (independent of BM) > 3 months. Exclusion criteria were: Small Cell Lung Cancer (SCLC), lymphoma, leukemia, leptomeningeal disease, contraindications for MRI or gadolinium contrast, and progressive symptomatic systemic disease without treatment options.

Additional eligibility criteria for study participation were: total tumor volume in the brain ≤ 30 cm^3^ (based on visual inspection of the MRI-scan) and age ≥ 18 years. Additional exclusion criteria were: a second active primary tumor, presence of an active primary brain tumor, prior brain radiation, prior brain surgery, severe cerebrovascular disease in the past 2 years, additional (history of a) significant neurological or psychiatric condition, participation in a concurrent study with neuropsychological testing and/or HRQoL assessments, comorbid medical condition precluding adequate follow-up, lack of basic proficiency in Dutch, IQ below 85, severe aphasia, severe visual problems, and paralysis of the hand(s)/arm(s).

### Procedure

During the first SRS consultation and information visit, the radiation-oncologist screened for SRS and study eligibility and provided patients with information about the study and its procedures. Eligible patients were treated within 1 week after the first consultation visit. If patients were willing to participate in the study, a neuropsychological assessment (NPA), including questionnaires concerning HRQoL, fatigue, and anxiety and depression, was administered by a trained test-leader (neuropsychologist or neuropsychologist in training) on the morning prior to SRS. Completion of the NPA and questionnaires took approximately 60 min. All patients signed for informed consent.

### Measures

HRQoL was measured with the Functional Assessment of Cancer Therapy-Brain (FACT-Br) [12]. The FACT-Br is a commonly used instrument to measure general HRQoL and specific symptoms or problems associated with brain tumors across five subscales, two total scores, and one index score (Table 1). Higher scores indicate a better HRQoL [12–15]. Published data from two normative samples provided by Brucker et al. [16] were used to compare pretreatment HRQoL of the patients with BM to. The first normative sample consisted of 1075 persons from the general U.S. adult population (age range = 18–91, 51% female). The second normative sample consisted of 2236 adult cancer patients (age range = 18–92, 57% female).

**Table 1:** Patient reported outcome measures

Questionnaires	Description	Scales/items
Functional Assessment of Cancer Therapy-Brain (FACT-Br)	The FACT-Br was developed for patients with primary brain tumors. Questions are answered on a 5-point Likert scale ranging from 0 (not at all) to 4 (very much), based on the past 7 days. Higher scores indicate a better HRQoL. The 5 subscales of the FACT-Br are focused on physical, social, emotional, and functional well-being, and additional concerns of patients with brain tumors. The FACT-General total score measures overall HRQoL and can be used in various groups of patients with cancer. In the FACT-Br total scale, a disease-specific subscale (i.e., *additional concerns*) is added to the FACT-General to measure HRQoL concerns specific to patients with a brain tumor. The trial outcome index combines physical well-being, functional well-being and the brain cancer subscale [12–15]. The FACT-Br has good internal consistency (.69 to .84) and reliability coefficients (.60 to .83) [14] and has been proven to be a valid HRQoL measure for use in patients with BM [13]	• Five subscales ◦ Physical well-being ◦ Social/family well-being ◦ Emotional well-being ◦ Functional well-being ◦ Brain cancer subscale (additional concerns specific for patients with brain tumors) • Two total scales ◦ FACT-General (FACT-G; physical + social + emotional + functional well-being) ◦ FACT-Brain (FACT-Br; FACT-G + brain cancer subscale) • One index ◦ Trial Outcome Index (TOI; physical + functional well-being + brain cancer subscale)
Hospital Anxiety and Depression Scale (HADS)	The HADS is a brief 14-item self-report measure consisting of seven anxiety items and seven depression items, measured on 4-point response scales (ranging from 0 to 3), referring to overt symptoms within the preceding week. Two subscales, anxiety and depression, can be calculated. A score ≥ 8 on each subscale is an indication for mild anxiety or depression [17, 33]	• Anxiety • Depression
Multidimensional Fatigue Inventory (MFI)	The MFI is a self-report instrument and consists of 20 items grouped in five dimensions each containing four items. The responder indicates on a five-point scale to what extent the statement applies to him or her based on the last couple of days. A higher score indicates more fatigue [18, 34]	• General fatigue • Physical fatigue • Reduced activity • Reduced motivation • Mental fatigue

*BM* brain metastases, *FACT*-*Br* Functional Assessment of Cancer Therapy-Brain, *FACT*-*G* FACT-General, *HADS* Hospital Anxiety and Depression Scale, *HRQoL* Health-Related Quality of Life, *MFI* Multidimensional Fatigue Inventory, *TOI* Trial Outcome Index

In addition, symptoms of anxiety and depression were measured with the Hospital Anxiety and Depression Scale (HADS) [17] and fatigue was measured with the Multidimensional Fatigue Inventory (MFI) [18] (Table 1).

For the current analyses, the cognitive factors immediate and delayed verbal memory (Hopkins Verbal Learning Test-Revised [19]), executive functioning (Trail Making Test [20]; the performance on part B given the performance on part A), and dominant and non-dominant motor dexterity (Grooved Pegboard [21]) were selected as (possible) cognitive predictors for HRQoL. Previous studies demonstrated that performances on these domains were most frequently impaired in patients with BM [4, 8, 22]. Raw cognitive test scores were converted into Z-scores based on our own normative group consisting of 104 Dutch non-cancer adults (see Online Resource 1 for a comparison of the characteristics of our patient sample and the non-cancer controls). Individual Z-scores were calculated using the following formula: Z-score = Y_o_ − Y_p_/SD_residual_, where Y_o_ is the raw cognitive test score of the individual, Y_p_ is the predicted raw cognitive test score using normative regression-based formulae (including age, sex and education as covariates resulting in sociodemographic adjusted norms), and SD_residual_ is the SD of the general population’s residual (see for example Rijnen et al. [23]).

Socio-demographic and clinical factors were retrieved from patient’s medical files (Table 2).

**Table 2:** Patient characteristics

	No. of patients (%)
Number of patients	92
Age in years, median (range)	63 (31–80)
Sex, male	47 (51.1)
Education^a^	
Low	28 (30.4)
Middle	37 (40.2)
High	27 (29.3)
Histology of the primary cancer	
Lung (NSCLC)	55 (59.8)
Renal	15 (16.3)
Melanoma	12 (13.0)
Breast	6 (6.5)
Other	4 (4.4)
Number of BM	
1	32 (34.8)
2–4	29 (31.5)
5–10	31 (33.7)
Total tumor volume cm^3^, median (range)	5.6 (0.02–31.1)
KPS, median (range)	90 (70–100)
RPA	
Class 1	16 (17.4)
Class 2	76 (82.6)
GPA	
Class 2	15 (16.3)
Class 3	60 (65.2)
Class 4	17 (18.5)
Seizures	
Yes	22 (23.9)
No	70 (76.1)
Diagnosis of BM	
Synchronous	28 (30.4)
Metachronous	64 (69.6)
Symptomatic BM	
Symptomatic	64 (69.6)
Asymptomatic	28 (30.4)
Symptoms of anxiety^b^	
No indication for anxiety	53 (57.6)
Indication for mild anxiety	39 (42.4)
Symptoms of depression^b^	
No indication for depression	62 (67.4)
Indication for mild depression	30 (32.6)

*BM* brain metastases, *GPA* graded prognostic assessment, *KPS* Karnofsky performance status, *No.* number, *NSCLC* non-small cell lung cancer, *RPA* recursive partitioning analysis

^a^The seven categories to classify the level of education of the Verhage scale [35] were merged into low (Verhage 1–4), middle (Verhage 5), and high (Verhage 6 and 7) educational level

^b^A score ≥ 8 per subscale is an indication for mild anxiety/depression [17]

### Statistical analyses

Individual raw HRQoL scores of the patients with BM were converted to T-scores (M = 50, standard deviation (SD) = 10), using the conversion tables based on data from the general U.S. adult population as provided by Brucker et al. [16]. A T-score of half a SD below the normative mean (T-score ≤ 45) was defined as clinically meaningful lower as compared to the general population [16]. The number of patients with a T-score ≤ 45 were counted to determine the prevalence of patients who scored clinically meaningful lower as compared to the general population.

One-sample *z* tests were conducted to investigate if there were statistical differences in mean HRQoL T-scores between patients with BM and the sample from the general population (T-score = 50, SD = 10) and between patients with BM and the adult cancer sample (again, T-score = 50, SD = 10) for all HRQoL subscales. As a measure of effect size, Glass’ Delta was calculated, by dividing the difference between the means of the groups by the standard deviation of the normative group for each FACT-Br subscale. An effect size ≤ 0.49 was considered a ‘small’ effect, from 0.50 to 0.79 a ‘medium’ effect and ≥ 0.80 a ‘large’ effect [24].

Meaningful differences, based on clinical and subjective indicators, were provided by Brucker et al. [16] as well. A mean difference of ≥ 2 points for the subscales physical, social, emotional and functional well-being and a mean difference of ≥ 5 points for general HRQoL were considered clinically meaningful.

Exploratory univariate linear regression analyses were performed in order to select candidate variables for use in the final multiple regression models. The univariate regression analyses included pretreatment socio-demographic (sex and age), clinical (KPS, RPA class, GPA class, total BM volume (cm^3^), (previous) seizures, symptomatic BM, illness duration (time in months between diagnosis of the primary cancer and SRS) and, synchronous versus metachronous diagnosis of BM (within or after 30 days of the diagnosis of the primary tumor, respectively), psychological (fatigue, depression and anxiety), and cognitive factors (immediate and delayed verbal memory, executive functioning and motor dexterity). For each factor and HRQoL subscale a separate regression analysis was run.

In a second step, the multiple regression analyses were run, for each HRQoL scale, in which all statistically significant factors (*p *< .05) of the initial linear regression analyses were included, resulting in eight regression models. If the assumption of homoscedasticity was violated, weighted least-squares (WLS) regressions were conducted. The absolute residuals were used as dependent variable in the multiple regression analyses to estimate the conditional error variances. Conditional error variances are used to calculate the weight variable, using the following formula: weight = 1/(conditional error variances^2^).

Statistical analyses were performed with SPSS version 24 (IBM Corp, 2016). A corrected alpha, based on the procedure of Benjamini–Hochberg [25], was used to reduce the false discovery rate due to multiple testing, separately for the one-samples z-tests, for the 8 multiple regression models and for the overall regression models.

## Results

Ninety-two patients with BM were included (Table 2). The median age of the patients was 63 years (range 31–80). Most frequent were a solitary BM (34.8%), primary Non-Small Cell Lung Cancer (NSCLC; 59.8%), and GPA class 3 (65.2%). The median total tumor volume in the brain was 5.6 cm^3^.

### Health-related quality of life

On the individual level, 64.1% of the patients had clinically meaningful low HRQoL (T-score ≤ 45) on at least one aspect of HRQoL as compared to the general adult population. The highest frequencies of low HRQoL scores were found for emotional well-being (57.6%), functional well-being (35.9%) and general HRQoL (32.6%) (Table 3).

**Table 3:** Percentages of patients with BM with low HRQoL and mean HRQoL scores of patients with BM compared to normative data of the general adult population and adult cancer sample

Normative data of the general population^a^	Percentage of patients scoring clinically meaningful lower compared to the general adult population (%)	Patients with BM (n = 92)				Patients with BM versus general population (n = 1075)^a^			
		Mean raw score	SD	Mean T-score	SD	Mean diff	z	*p* ^b^	Effect size^c^
Physical well-being	27.2	22.7	4.8	50.0	8.9	0.0	0.0	1.00	.00
Social well-being	7.6	23.0	5.3	55.6	7.8	5.6	5.4	**< .001**	.56
Emotional well-being	57.6	16.0	4.7	41.9	9.8	− 8.1	− 7.8	**< .001**	.81
Functional well-being	35.9	17.9	6.1	49.1	8.9	− 0.9	− 0.9	.39	.09
FACT-General	32.6	79.6	15.6	49.7	8.6	− 0.3	− 0.3	.77	.03
Additional concerns^d^		50.5	11.2						
FACT-Brain^d^		130.1	24.0						
Trial outcome index^d^		91.1	18.8						

Normative data of adult cancer sample^e^	Patients with BM (n = 92)		Patients with BM versus adult cancer sample (n = 2236)^e^			
	Mean T-score	SD	Mean diff	z	*p* ^b^	Effect size^c^
Physical well-being	52.3	8.0	2.3	2.2	.03	.23
Social well-being	51.5	10.0	1.5	1.4	.15	.15
Emotional well-being	44.0	10.4	− 6.0	− 5.8	**< .001**	.60
Functional well-being	48.5	8.9	− 1.5	− 1.4	.15	.15
FACT-general	49.2	9.2	− 0.8	− 0.8	.44	.08

Bold text indicates a statistically significant difference

*BM* brain metastases, *FACT* Functional assessment of cancer therapy, *HRQoL* health-related quality of life, *mean diff* mean difference, *n* number of participants, *SD* standard deviation

^a^Normative data of general population (n = 1075) of Brucker et al. [16], mean 50; SD 10

^b^Corrected alpha of .015, using the Benjamini-Hochberg procedure [25]

^c^Glass’s Delta

^d^Normative data not available

^e^Normative data of adult cancer sample (n = 2236) of Brucker et al. [16], mean 50; SD 10

At the level of group means, patients with BM, as compared to the general population [16], had a significantly and clinically meaningful (e.g. > 2 points difference) better social well-being and worse emotional well-being (Table 3). Compared to the adult cancer sample [16], patients with BM had significantly and clinically meaningful lower mean scores on emotional well-being (Table 3). Although there was no statistically significant difference on physical well-being between patients with BM and the adult cancer sample, patients with BM reported a clinically meaningful better physical well-being compared to the adult cancer sample.

### Predictors of baseline health-related quality of life

Results of the exploratory univariate analyses are presented in the supplementary Online Resource 2. Factors selected for subsequent multiple regression analyses are presented in Table 4. Due to multicollinearity between the subscales of the MFI, general fatigue and reduced activity were excluded (each had a high correlation with physical fatigue, r = .866 and r = .736, respectively).

**Table 4:** Multivariate analyses of predictors of pre-SRS HRQoL

		PWB^a^	SWB^a^	EWB	FWB
Model summary					
F		26.775	24.903	25.955	19.268
*Adjusted R*^2^		.723	.570	.526	.552
*p* value*		**< .001**	**< .001**	**< .001**	**< .001**
n		90	91	91	90
*Clinical factors*					
KPS 70–80 versus 90–100 (ref)	b	− 0.077	NS	NS	− 0.290
	95% CI	− 1.093 to 0.939			− 2.372 to 1.791
	*p* value^*^	.881			.782
Seizures Yes versus No (ref)	b	NS	− 1.848	NS	NS
	95% CI		− 4.734 to 1.038		
	*p* value^*^		.206		
RPA Class 1 versus Class 2 (ref)	b	− 1.269	NS	NS	NS
	95% CI	− 3.676 to 1.139			
	*p* value^*^	.297			
Illness duration	b	− 0.016	NS	NS	NS
	95% CI	− 0.034 to 0.002			
	*p* value^*^	.088			
Diagnosis of BM Metachronous versus synchronous (ref)	b	− 0.604	− 2.863	NS	NS
	95% CI	− 1.676 to 0.469	− 4.554 to − 1.173		
	*p* value^*^	.266	**.001**		
*Psychological factors*					
Physical fatigue	b	− 0.409	NS	− 0.087	− 0.446
	95% CI	− 0.543 to − 0.275		− 0.279 to 0.105	− 0.699 to − 0.192
	*p* value^*^	**< .001**		.372	**.001**
Reduced motivation	b	− 0.020	0.251	− 0.197	− 0.282
	95% CI	− 0.170 to 0.131	0.183 to 0.319	− 0.448 to 0.053	− 0.594 to 0.031
	*p* value^*^	.796	**< .001**	.121	.077
Mental fatigue	b	− 0.175	NS	NS	0.081
	95% CI	− 0.290 to − 0.061			− 0.158 to 0.320
	*p* value^*^	**.003**			.500
Anxiety	b	− 0.074	NS	− 0.629	− 0.004
	95% CI	− 0.223 to 0.075		− 0.834 to − 0.425	− 0.270 to 0.262
	*p* value^*^	.324		**< .001**	.975
Depression	b	− 0.223	− 0.289	− 0.080	− 0.645
	95% CI	− 0.418 to − 0.028	− 0.507 to − 0.072	− 0.320 to 0.160	− 0.970 to − 0.320
	*p* value^*^	.025	**.010**	.510	**< .001**
*Cognitive factors*					
Immediate verbal memory	b	NS	0.003	NS	NS
	95% CI		− 0.470 to 0.476		
	*p* value^*^		.990		
Motor dexterity Dominant hand	b	NS	NS	NS	NS
	95% CI				
	*p* value^*^				
Motor dexterity Non- dominant hand	b	NS	NS	NS	NS
	95% CI				
	*p* value^*^				

		FACT-G	BRCS	FACT-Br	TOI
Model summary					
F		22.066	9.353	20.990	21.351
*Adjusted R*^2^		.624	.475	.684	.688
*p* value*		**< .001**	**< .001**	**< .001**	**< .001**
n		90	84	84	84
*Clinical factors*					
KPS 70–80 versus 90–100 (ref)	b	0.707	− 1.969	− 1.613	− 1.800
	95% CI	− 4.288 to 5.702	− 6.914 to 2.975	− 9.619 to 6.394	− 8.070 to 4.470
	*p* value^*^	.779	.430	.689	.569
Seizures Yes versus No (ref)	b	NS	NS	NS	NS
	95% CI				
	*p* value^*^				
RPA Class 1 versus Class 2 (ref)	b	NS	NS	NS	NS
	95% CI				
	*p* value^*^				
Illness duration	b	NS	NS	NS	NS
	95% CI				
	*p* value^*^				
Diagnosis of BM Metachronous versus synchronous (ref)	b	− 3.931	− 1.169	− 6.640	− 3.257
	95% CI	− 8.649 to 0.788	− 5.565 to 3.227	− 13.758 to 0.479	− 8.831 to 2.317
	*p* value^*^	.101	.598	.067	.248
*Psychological factors*					
Physical fatigue	b	− 1.054	− 0.490	− 1.272	− 1.303
	95% CI	− 1.646 to − 0.461	− 1.078 to 0.097	− 2.224 to − 0.321	− 2.048 to − 0.558
	*p* value^*^	**.001**	.101	**.009**	**.001**
Reduced motivation	b	− 0.765	− 0.025	− 1.067	− 0.383
	95% CI	− 1.495 to − 0.035	− 0.730 to 0.679	− 2.208 to 0.074	− 1.276 to 0.511
	*p* value^*^	.040	.943	.066	.396
Mental fatigue	b	0.314	− 0.733	− 0.077	− 0.597
	95% CI	− 0.264 to 0.892	− 1.303 to − 0.163	− 1.000 to 0.846	− 1.320 to 0.125
	*p* value^*^	.283	.012	.869	.104
Anxiety	b	− 0.725	− 0.043	− 0.854	− 0.061
	95% CI	− 1.354 to − 0.097	− 0.662 to 0.577	− 1.858 to 0.149	− 0.847 to 0.725
	*p* value^*^	.024	.892	.094	.878
Depression	b	− 1.356	− 0.938	− 2.526	− 2.238
	95% CI	− 2.117 to − 0.596	− 1.678 to − 0.197	− 3.725 to − 1.327	− 3.177 to − 1.299
	*p* value^*^	**.001**	.014	**< .001**	**< .001**
*Cognitive factors*					
Immediate verbal memory	b	NS	NS	NS	NS
	95% CI				
	*p* value^*^				
Motor dexterity Dominant hand	b	NS	0.705	1.265	0.795
	95% CI		− 0.004 to 1.415	0.116 to 2.414	− 0.105 to 1.694
	*p* value^*^		.051	.031	.083
Motor dexterity Non- dominant hand	b	NS	0.145	− 0.029	0.022
	95% CI		− 0.836 to 1.126	− 1.618 to 1.560	− 1.222 to 1.266
	*p* value^*^		.769	.971	.972

Bold text indicates a statistically significant result

*BRCS* brain cancer subscale, *CI* confidence interval, *EWB* emotional well-being, *FACT*-*Br* functional assessment of cancer therapy-Brain, *FACT*-*G* FACT-General, *FWB* functional well-being, *HRQoL* health-related quality of life, *KPS* Karnofsky performance status, *n* number of patients, *NS* not selected based on univariate analyses (*p* ≥ .05), *PWB* physical well-being, ref: reference category, *RPA* recursive partitioning analysis, *SWB* social well-being, *TOI* trial outcome index

*Corrected alpha’s, following the Benjamini-Hochberg procedure [25], were .05 for the overall models, .011 for PWB, .01 for SWB, .008 for EWB, .017 for FWB, .014 for FACT-G, .006 for BRCS, and .011 for FACT-Br and TOI. Not selected factors: sex, age, GPA class, total BM volume (cm^3^), symptomatic BM, delayed verbal memory, and executive functioning

^a^Weighted least squares regression

All regression models significantly predicted HRQoL (*p* ≤ .001) (Table 4). Better physical well-being was associated with lower levels of physical and mental fatigue. Better social well-being was associated with a synchronous diagnosis of BM, lower levels of reduced motivation, and fewer symptoms of depression. Better emotional well-being was associated with fewer symptoms of anxiety. Better functional well-being and general HRQoL were associated with lower levels of physical fatigue and fewer symptoms of depression. Better FACT-Brain and trial outcome index scores were associated with lower levels of physical fatigue and fewer symptoms of depression. None of the factors added significantly to the prediction of additional concerns.

## Discussion

On the individual level, 64.1% of the patients with BM already reported clinically meaningful low HRQoL on at least one subscale just before treatment, compared to the general population, of whom most patients (57.6%) reported problems with emotional well-being. Van der Meer et al. [9] reported that 89% of patients had a significant impairment on at least one subscale of The European Organization for Research and Treatment of Cancer Quality of Life Questionnaire Core 30 (EORTC-QLQ-C30), and the most frequently affected aspect of HRQoL was physical functioning (57%) [9]. Both studies indicate that the majority of patients already experience problems with HRQoL pre-SRS.

On the group level, our patients with BM reported statistically significant and clinically meaningful lower emotional well-being than the general population and adult cancer sample. The difference with other cancer patients may be explained by the emotional distress caused by a more recent diagnosis of a serious life-threatening disease and the upcoming treatment. In addition, our patients with BM probably were in a more advanced disease stage compared to the adult cancer sample and might therefore experience more emotional distress. Patients with BM reported significantly, and clinically meaningful, higher social well-being compared to the general population, which could be explained by the increase of support that patients receive just before the upcoming treatment. Habets et al. [8], evaluating group results in the same patient sample as van der Meer et al. [9], also found worse emotional functioning pre-SRS as compared to the general population, but their patients with BM also scored significantly worse on physical functioning and global health status/QoL, which was not found in the current study. Our patients with BM scored clinically meaningful better on physical well-being than the adult cancer sample, although this was not statistically significant.

A possible explanation for the differences between the results of the current study and those of the studies of Habets et al. [8] and van der Meer et al. [9], is the difference in questionnaires that were used to measure HRQoL. The EORTC-QLQ-C30 used by Habets et al. [8] and van der Meer et al. [9] is known to be more focused on functional *activities*, while the FACT-Br used in the current study is more focused on functional *symptoms* [26, 27]. Patients with BM might experience or report more problems with functional activities, such as taking a long walk, than with functional symptoms, such as pain.

The before mentioned explanations for differences in HRQoL between the patients with BM and the adult cancer sample are primarily based on the moment at which the patients with BM completed the HRQoL questionnaire. The pretreatment HRQoL measurement is therefore very important when analyzing change in HRQoL over time, as changes over time in HRQoL could not only be due to treatment-related factors, but also due to factors that are already present at the pretreatment measurement. It is therefore advisable to interpret HRQoL results over time only after careful evaluation of HRQoL before treatment.

Overall, worse psychological factors, e.g. physical fatigue, depression, mental fatigue and anxiety, were predictive for lower pre-SRS HRQoL. Patients may experience additional anxiety or depression on the day of treatment, resulting in lower HRQoL. Pre-SRS, 42.4% and 32.6% of the patients scored above the cutoff for (at least) mild symptoms of anxiety and depression respectively. Clinicians and nursing staff should, if possible, pay extra attention to patients that experience these psychological complaints. A synchronous (as opposed to a metachronous) diagnosis of BM predicted better social well-being. This might be due to increased social support after a first-time diagnosis of a life-threatening disease. Pre-SRS KPS did not predict HRQoL of patients with BM on any of the subscales, whereas in a previous study, using univariate analyses, pre-SRS KPS was associated with pre-SRS HRQoL [8]. In the univariate linear regression analyses of the current study KPS was also a significant positive predictor for 6 of the 8 HRQoL scales, however KPS was no longer a significant predictor in the multiple regression analyses. Objectively tested cognitive performance did not predict any of the pretreatment aspects of HRQoL, whereas the absence of cognitive symptoms, as measured by self-report, was a significant predictor for HRQoL after SRS in the study of Skeie et al. [11]. This might be due to differences in measuring cognitive functioning; self-reported cognitive functioning might be more related to self-reported HRQoL than objectively tested cognitive functioning [28].

It should be noted that in this study a subset of patients with BM, eligible for GKRS and able and willing to complete an NPA, was included. Overall, patients with BM selected for GKRS have a relatively good clinical condition. In addition, a more resilient group of patients may have been included in this study compared to other HRQoL studies with less time-consuming and burdensome assessments (patients were also asked to complete several neuropsychological tests). Lastly, a heterogeneous study sample of patients with several types of primary cancers was included. Different primary tumors may have influenced aspects of HRQoL differently (e.g. due to their different symptoms or side effects). However, the study sample as a whole is representative for the group of patients with BM that is generally treated with GKRS in our center.

For patients with BM, HRQoL is a highly important factor in choosing among treatment options [5]. In future studies, HRQoL of patients with BM from different primary tumors may be analyzed separately. In addition, all aspects of HRQoL should be evaluated separately, as our findings show different outcomes for the different aspects of HRQoL and combining scores into a total score may potentially mask specific problems with aspects of HRQoL. Furthermore, the pre-SRS HRQoL state of patients should be analyzed before results on change over time in HRQoL are interpreted.

Since increasingly more patients with BM are being treated with SRS [6, 7], our findings are very relevant to a large group of patients with BM. There should be more awareness of the well-being of patients with BM before treatment; especially with respect to the low emotional well-being. Clinicians and nursing staff should be better informed on the pre-SRS HRQoL states of patients and the specific issues they are dealing with, and thereby better understand a patient’s feelings and needs on the day of the treatment. Standard assessment of HRQoL in the clinical practice is helpful to identify patients’ concerns [29], may improve communication between patients and clinicians, helps clinicians to provide patients with personalized information, and guide, if necessary, decisions on interventions for HRQoL problems after SRS. Interventions that have been found helpful for improving HRQoL include cognitive behavioral therapy or psychotherapy [30–32]. These interventions also aim at improving symptoms of anxiety and depression. Our findings indicated that it is important to address these psychological factors when aiming for improvement of HRQoL in patients with BM.

## Electronic supplementary material

Below is the link to the electronic supplementary material.

Supplementary material 1 (PDF 97 kb)

Supplementary material 2 (PDF 134 kb)
