# Supplementary material for: Health-related quality of life of patients with brain metastases selected for stereotactic radiosurgery
Source: J Neurooncol. 2019 May 9;143(3):537–46. doi: 10.1007/s11060-019-03186-z (PMC6591192; doi:10.1007/s11060-019-03186-z)
Supplement: Supplementary file 1 — Supplementary material 1 (PDF 97 kb) [file 11060_2019_3186_MOESM1_ESM.pdf]

Journal of Neuro-Oncology – Online Resource 1

Health-related quality of life of patients with brain metastases selected for stereotactic radiosurgery

Eline Verhaak[1,2,3], Karin Gehring[1,2,3], Patrick E. J. Hanssens[1,2], AND Margriet M. Sitskoorn[2,3]
[1] Gamma Knife Center, Elisabeth-TweeSteden Hospital, Tilburg, The Netherlands
[2] Department of Neurosurgery, Elisabeth-TweeSteden Hospital, Tilburg, The Netherlands
[3] Department of Cognitive Neuropsychology, Tilburg University, Tilburg, The Netherlands

Corresponding author: Karin Gehring (E-mail: k.gehring@tilburguniversity.edu, Telephone number: +31 13 466 4233)

Non-cancer controls – CAR-Study A (NCT02953756)

Dutch non-cancer controls were recruited by convenience sampling of the broad network of the research group. Inclusion criteria were; age ≥18 years, no (history of) cancer and no cerebrovascular disease in the past 12 months. All controls signed informed consent. Characteristics of the control group are presented in Table 1.

Table 1. Characteristics

	No. of patients with BM (%)	No. of controls (%)	t-score	χ^2	P^*
**Number of participants**	92	104			
**Age in years,** median (range)	63 (31-80)	60 (31-87)	1.527		.128
**Sex**, male	47 (51)	50 (48)		0.177	.674
**Education**[a]				4.626	.099
Low	28 (30)	25 (24)			
Middle	37 (40)	33 (31)			
High	27 (29)	46 (44)			

Note: No.: number, percentages have been rounded and may not total to 100%
[a] The 7 categories to classify the level of education of the Verhage scale [1] were merged into low (Verhage 1 – 4), middle (Verhage 5), and high (Verhage 6 and 7) educational level
[*] Corrected alpha of .02, using the Benjamini-Hochberg procedure [2]

Neuropsychological assessments were administered at baseline, and at 3 and 6 months thereafter. It took approximately 60 minutes to complete. The neuropsychological test battery included 6 short cognitive tests; Hopkins Verbal Learning Test-Revised, Trail Making Test, Controlled Oral Word Association, Wechsler Adult Intelligence Scale (WAIS) Digit Span, WAIS Digit Symbol and Grooved Pegboard [3-5].

References

1. Verhage, F. (1964). *Intelligentie en leeftijd: onderzoek bij Nederlanders van twaalf tot zevenenzeventig jaar.* Assen, The Netherlands: Van Gorcum.
2. Benjamini, Y., & Hochberg, Y. (1995). Controlling the false discovery rate: a practical and powerful approach to multiple testing. *Journal of the royal statistical society. Series B (Methodological)*, 289-300.
3. Wefel, J. S., Vardy, J., Ahles, T., & Schagen, S. B. (2011). International Cognition and Cancer Task Force recommendations to harmonise studies of cognitive function in patients with cancer. *The lancet oncology, 12*(7), 703-708.
4. Witgert, M. E., & Meyers, C. A. (2011). Neurocognitive and quality of life measures in patients with metastatic brain disease. *Neurosurgery Clinics, 22*(1), 79-85.
5. Meyers, C. A., & Brown, P. D. (2006). Role and relevance of neurocognitive assessment in clinical trials of patients with CNS tumors. *Journal of clinical oncology, 24*(8), 1305-1309.
