# Supplementary material for: Health-related quality of life of patients with brain metastases selected for stereotactic radiosurgery
Source: J Neurooncol. 2019 May 9;143(3):537–46. doi: 10.1007/s11060-019-03186-z (PMC6591192; doi:10.1007/s11060-019-03186-z)
Supplement: Supplementary file 2 — Supplementary material 2 (PDF 134 kb) [file 11060_2019_3186_MOESM2_ESM.pdf]

Journal of Neuro-Oncology – Online Resource 2

Health-related quality of life of patients with brain metastases selected for stereotactic radiosurgery

Eline Verhaak[1,2,3], Karin Gehring[1,2,3], Patrick E. J. Hanssens[1,2], AND Margriet M. Sitskoorn[2,3]
[1] Gamma Knife Center, Elisabeth-TweeSteden Hospital, Tilburg, The Netherlands
[2] Department of Neurosurgery, Elisabeth-TweeSteden Hospital, Tilburg, The Netherlands
[3] Department of Cognitive Neuropsychology, Tilburg University, Tilburg, The Netherlands

Corresponding author: Karin Gehring (E-mail: k.gehring@tilburguniversity.edu, Telephone number: +31 13 466 4233)

Results of the exploratory univariate analyses of predictors of pre-SRS HRQoL of patients with BM – CAR-Study A (NCT02953756)

Supplementary Table 1. Univariate analyses of predictors of pre-SRS HRQoL of 92 patients with BM

		**PWB**	**SWB**	**EWB**	**FWB**	**FACT-G**	**BRCS**	**FACT-Br**	**TOI**
**Socio-demographic factors**									
Age	b	-0.004	-0.063[¥]	0.012	-0.037	-0.081	-0.148	-0.229	-0.188
	95% CI	-0.107 to 0.099	-0.170 to 0.044	-0.089 to 0.113	-0.168 to 0.093	-0.417 to 0.255	-0.387 to 0.092	-0.743 to 0.286	-0.592 to 0.215
	p value	.943	.243	.811	.573	.634	.224	.380	.355
	Adjusted R^2	-.011	.004	-.010	-.008	-.009	.005	-.002	-.002
Sex	b	-0.880	1.150	-1.870	-0.656	-2.257	-1.981	-4.237	-3.516
Female vs Male (ref)	95% CI	-2.865 to 1.106	-1.047 to 3.347	-3.785 to -0.044	-3.179 to 1.867	-8.747 to 4.234	-6.630 to 2.669	-14.196 to 5.722	-11.314 to 4.281
	p value	.381	.301	.055	.607	.492	.400	.400	.373
	Adjusted R^2	-.002	.001	.030	-.008	-.006	-.003	-.003	-.002
**Clinical factors**									
KPS	b	-3.405	-2.010	-1.418	-4.968	-11.800	-9.574	-21.374	-17.947
70 - 80 vs 90 - 100 (ref)	95% CI	-5.357 to -1.453	-4.275 to 0.255	-3.433 to 0.597	-7.387 to -2.548	-18.117 to -5.484	-14.007 to -5.141	-30.785 to -11.963	-25.194 to -10.700
	p value	**.001**	.081	.166	**<.001**	**<.001**	**<.001**	**<.001**	**<.001**
	Adjusted R^2	.108	.023	.010	.147	.123	.161	.175	.203
Total BM volume	b	-0.011	-0.025	0.024	-0.027	-0.039	-0.098	-0.137	-0.136
	95% CI	-0.142 to 0.121	-0.171 to 0.121	-0.105 to 0.152	-0.194 to 0.139	-0.468 to 0.390	-0.405 to 0.209	-0.796 to 0.522	-0.652 to 0.380
	p value	.873	.735	.715	.748	.858	.527	.680	.602
	Adjusted R^2	-.011	-.010	-.010	-.010	-.011	-.007	-.009	-.008
Seizures	b	0.104	-2.720	0.000	-1.558	-4.174	-3.510	-7.684	-4.965
Yes vs No (ref)	95% CI	-2.233 to 2.440	-5.247 to -0.193	-2.290 to 2.290	-4.502 to 1.385	-11.750 to 3.402	-8.931 to 1.911	-19.291 to 3.922	-14.085 to 4.155
	p value	.930	**.035**	1.000	.296	.277	.202	.192	.282
	Adjusted R^2	-.011	.038	-.011	.001	.002	.007	.008	.002

		PWB	SWB	EWB	FWB	FACT-G	BRCS	FACT-Br	TOI
RPA	b	-3.942	0.535	-0.832	-2.013	-6.252	-3.378	-9.631	-9.333
Class 1 vs Class 2 (ref)	95% CI	-6.439 to -1.445	-2.378 to 3.448	-3.404 to 1.739	-5.319 to 1.293	-14.734 to 2.230	-9.494 to 2.737	-22.662 to 3.401	-19.477 to 0.810
	p value	**.002**	.716	.522	.230	.147	.275	.146	.071
	Adjusted R^2	.089	-.010	-.006	.005	.012	.002	.013	.025
GPA									
Class 2 vs Class 4 (ref)	b	0.824	2.129	2.294	3.537	8.784	0.245	9.029	4.606
	95% CI	-2.569 to 4.217	-1.620 to 5.877	-1.007 to 5.595	-0.712 to 7.787	-2.176 to 19.744	-7.729 to 8.219	-7.943 to 26.002	-8.732 to 17.945
Class 3 vs Class 4 (ref)	b	-0.315	1.207	1.411	1.237	3.540	0.030	3.570	0.953
	95% CI	-2.947 to 2.317	-1.701 to 4.114	-1.150 to 3.971	-2.059 to 4.533	-4.961 to 12.040	-6.154 to 6.215	-9.594 to 16.734	-9.393 to 11.298
	p value	.715	.523	.370	.248	.282	.998	.570	.758
	Adjusted R^2	-.015	-.008	.000	.009	.006	-.022	-.010	-.016
Symptomatic BM	b	-0.081	-0.918	-0.051	-1.614	-2.664	-3.676	-6.339	-5.370
Yes vs No (ref)	95% CI	-2.247 to 2.085	-3.311 to 1.476	-2.174 to 2.072	-4.338 to 1.111	-9.711 to 4.384	-8.688 to 1.337	-17.120 to 4.442	-13.805 to 3.064
	p value	.941	.448	.962	.242	.455	.149	.246	.209
	Adjusted R^2	-.011	-.005	-.011	.004	-.005	.012	.004	.007
Illness duration	b	-0.028	-0.008	0.001	-0.015	-0.050	-0.014	-0.065	-0.058
	95% CI	-0.056 to 0.000	-0.040 to 0.024	-0.027 to 0.029	-0.051 to 0.021	-0.143 to 0.043	-0.081 to 0.053	-0.208 to 0.079	-0.170 to 0.054
	p value[*]	**.048**	.619	.948	.411	.285	.676	.373	.310
	Adjusted R^2	.032	-.008	-.011	-.003	.002	-.009	-.002	.000
Diagnosis of BM	b	-2.947	-2.932	0.051	-1.871	-7.698	-6.807	-14.506	-11.625
Metachronous vs	95% CI	-5.024 to -0.871	-5.253 to -0.610	-2.072 to 2.174	-4.588 to 0.847	-14.582 to -0.815	-11.674 to -1.941	-24.935 to -4.077	-19.779 to -3.471
Synchronous (ref)	*p* value	**.006**	**.014**	.962	.175	**.029**	**.007**	**.007**	**.006**
	Adjusted R^2	.071	.055	-.011	.009	.041	.069	.068	.072
**Psychological factors**									
General Fatigue	b	-0.705[¥]	-0.216	-0.390	-0.822	-2.094	-1.114	-3.208	-2.602
	95% CI	-0.861 to -0.549	-0.469 to 0.037	-0.602 to -0.178	-1.061 to -0.584	-2.712 to -1.476	-1.605 to -0.623	-4.159 to -2.257	-3.334 to -1.871
	p value	**<.001**	.094	**<.001**	**<.001**	**<.001**	**<.001**	**<.001**	**<.001**
	Adjusted R^2	.466	.020	.119	.335	.327	.175	.325	.350
Physical Fatigue	b	-0.660	-0.196	-0.377	-0.787	-2.020	-1.167	-3.188	-2.615
	95% CI	-0.826 to -0.494	-0.432 to 0.040	-0.574 to -0.181	-1.006 to -0.568	-2.585 to -1.456	-1.610 to -0.724	-4.042 to -2.334	-3.263 to -1.966
	p value	**<.001**	.102	**<.001**	**<.001**	**<.001**	**<.001**	**<.001**	**<.001**
	Adjusted R^2	.402	.019	.129	.355	.353	.225	.373	.409
Reduced Activity	b	-0.652	-0.334	-0.468	-0.934	-2.388	-1.396	-3.784	-2.982
	95% CI	-0.859 to -0.445	-0.599 to -0.068	-0.691 to -0.246	-1.180 to -0.688	-3.024 to -1.753	-1.897 to -0.895	-4.741 to -2.827	-3.729 to -2.235
	p value	**<.001**	**.014**	**<.001**	**<.001**	**<.001**	**<.001**	**<.001**	**<.001**
	Adjusted R^2	.296	.054	.154	.381	.376	.246	.400	.405
Reduced Motivation[a]	b	-0.643	-0.384	-0.506	-0.967	-2.501	-1.372	-3.873	-2.982
	95% CI	-0.874 to -0.412	-0.670 to -0.098	-0.745 to -0.268	-1.238 to -0.696	-3.197 to -1.805	-1.930 to -0.814	-4.941 to -2.805	-3.828 to -2.137
	p value	**<.001**	**.009**	**<.001**	**<.001**	**<.001**	**<.001**	**<.001**	**<.001**
	Adjusted R^2	.248	.064	.157	.353	.357	.203	.361	.348

		PWB	SWB	EWB	FWB	FACT-G	BRCS	FACT-Br	TOI
Mental Fatigue[a]	b	-0.485	-0.135	-0.202	-0.437	-1.259	-1.530	-2.789	-2.452
	95% CI	-0.712 to -0.258	-0.408 to 0.137	-0.447 to 0.043	-0.738 to -0.137	-2.016 to -0.503	-2.025 to -1.035	-3.884 to -1.695	-3.284 to -1.621
	p value	**<.001**	.326	.105	**.005**	**.001**	**<.001**	**<.001**	**<.001**
	Adjusted R^2	.159	.000	.018	.076	.099	.290	.215	.270
Anxiety	b	-0.449	-0.189	-0.749	-0.575	-1.963	-1.028	-2.991	-2.053
	95% CI	-0.659 to -0.240	-0.441 to 0.063	-0.911 to -0.588	-0.841 to -0.310	-2.590 to -1.336	-1.521 to -0.535	-3.958 to -2.024	-2.847 to -1.259
	p value	**<.001**	.139	**<.001**	**<.001**	**<.001**	**<.001**	**<.001**	**<.001**
	Adjusted R^2	.158	.013	.480	.162	.293	.151	.288	.218
Depression	b	-0.730[¥]	-0.340	-0.638	-0.969	-2.671	-1.558	-4.230	-3.251
	95% CI	-0.932 to -0.528	-0.601 to -0.079	-0.837 to -0.440	-1.202 to -0.735	-3.239 to -2.104	-2.028 to -1.089	-5.075 to -3.385	-3.927 to -2.576
	p value	**<.001**	**.011**	**<.001**	**<.001**	**<.001**	**<.001**	**<.001**	**<.001**
	Adjusted R^2	.357	.059	.305	.424	.487	.319	.518	.498
Cognitive factors									
Immediate verbal memory	b	-0.214	0.969[¥]	-0.355	0.305	0.631	1.007	1.639	1.098
	95% CI	-0.930 to 0.502	0.432 to 1.505	-1.054 to 0.344	-0.602 to 1.211	-1.705 to 2.968	-0.658 to 2.672	-1.940 to 5.218	-1.709 to 3.905
	p value	.554	**.001**	.316	.506	.593	.233	.365	.439
	Adjusted R^2	-.007	.115	.000	-.006	-.008	.005	-.002	-.004
Delayed verbal memory	b	-0.035	0.724[¥]	-0.388	0.259	0.322	1.277	1.599	1.501
	95% CI	-0.823 to 0.752	-0.169 to 1.617	-1.155 to 0.380	-0.737 to 1.256	-2.247 to 2.890	-0.547 to 3.101	-2.336 to 5.533	-1.576 to 4.578
	p value	.929	.111	.318	.607	.804	.168	.422	.335
	Adjusted R^2	-.011	.017	.000	-.008	-.010	.010	-.004	-.001
Executive functioning[b]	b	-0.059	0.126	-0.147	-0.104	-0.185	0.175	-0.010	0.012
	95% CI	-0.505 to 0.386	-0.363 to 0.615	-0.557 to 0.262	-0.657 to 0.449	-1.607 to 1.237	-0.842 to 1.193	-2.200 to 2.180	-1.711 to 1.735
	p value	.791	.610	.477	.709	.796	.733	.993	.989
	Adjusted R^2	-.012	-.009	-.006	-.011	-.012	-.011	-.013	-.013
Motor dexterity	b	0.098	0.320	0.250	0.279	0.947	1.275	2.222	1.652
Dominant hand[c]	95% CI	-0.268 to 0.463	-0.082 to 0.721	-0.089 to 0.589	-0.176 to 0.734	-0.222 to 2.116	0.465 to 2.085	0.461 to 3.982	0.266 to 3.037
	p value	.596	.117	.146	.226	.111	**.002**	**.014**	**.020**
	Adjusted R^2	-.008	.017	.013	.006	.018	.092	.057	.050
Motor dexterity	b	0.108	0.474	0.059	0.380	1.021	1.383	2.404	1.871
Non-dominant hand[c]	95% CI	-0.364 to 0.580	-0.041 to 0.989	-0.404 to 0.523	-0.202 to 0.961	-0.489 to 2.531	0.333 to 2.434	0.123 to 4.685	0.089 to 3.653
	p value	.650	.071	.800	.198	.183	**.010**	**.039**	**.040**
	Adjusted R^2	-.009	.026	-.011	.008	.009	.063	.038	.037

Note: [¥] Weighted Least Squares regression, [a] n=91, [b] n=80, [c] n=88

BM: brain metastases, BRCS: brain cancer subscale, CI: confidence interval, EWB: Emotional well-being, FACT-Br: Functional Assessment of Cancer Therapy-Brain, FACT-G: FACT-General, FWB: Functional well-being, GPA: Graded Prognostic Assessment, HRQoL: Health-related quality of life, KPS: Karnofsky performance status, PWB: Physical well-being, ref: reference category, RPA: Recursive Partitioning Analysis, SWB: Social well-being, TOI: trial outcome index, vs: versus
